# Using the *Culex pipiens* sperm proteome to identify elements essential for mosquito reproduction

**DOI:** 10.1371/journal.pone.0280013

**Published:** 2023-02-16

**Authors:** Catherine D. Thaler, Kaira Carstens, Gabrielle Martinez, Kimberly Stephens, Richard A. Cardullo

**Affiliations:** 1 Department of Evolution, Ecology and Organismal Biology, University of California, Riverside, Riverside, CA, United States of America; 2 Department of Biochemistry, University of California, Riverside, Riverside, CA, United States of America; 3 Department of Entomology, University of California, Riverside, Riverside, CA, United States of America; Cornell University College of Veterinary Medicine, UNITED STATES

## Abstract

Mature sperm from *Culex pipiens* were isolated and analyzed by mass spectrometry to generate a mature sperm proteome dataset. In this study, we highlight subsets of proteins related to flagellar structure and sperm motility and compare the identified protein components to previous studies examining essential functions of sperm. The proteome includes 1700 unique protein IDs, including a number of uncharacterized proteins. Here we discuss those proteins that may contribute to the unusual structure of the *Culex* sperm flagellum, as well as potential regulators of calcium mobilization and phosphorylation pathways that regulate motility. This database will prove useful for understanding the mechanisms that activate and maintain sperm motility as well as identify potential molecular targets for mosquito population control.

## Introduction

Sperm are terminally differentiated cells that lack protein synthetic machinery and are thereby constrained to function with a defined, and limited, array of proteins. This imposes specific constraints upon these cells. In particular, with limited energy stores, sperm are usually quiescent until signals to activate motility are received. Thus, while many sperm proteins are components of the eukaryotic 9+2 axoneme that powers the sperm flagellum and constitute a known set of proteins, the proteins that regulate flagellar motility in this non-regenerative system may differ from other cells that use cilia or flagella for motility. Moreover, motility regulators may vary across species depending upon the particular motility behaviors exhibited by the sperm. Some insect sperm, reportedly those with accessory microtubules surrounding the axoneme (the 9+9+2 axoneme), exhibit unusual waveforms when motile [1].

Sperm from species of Culicidae that have been examined to date, including *Culex pipiens* and *Culex quinquefasciatus*, possess a 9+9+1 axoneme in which the central pair of microtubules is replaced by a single central “rod” [2–4]. *Culex spp*. sperm exhibit the unusual waveforms described in other insect sperm: a double waveform flagellar beating pattern (a low amplitude, short wavelength wave superimposed upon a high amplitude, long wavelength), a single helical waveform with forward progressive motility, and, interestingly, the ability to swim backwards (progressive motility with head trailing) [5]. Given that the major structural features of the axoneme are highly conserved across eukaryotes, it is likely that particular regulatory components govern the ability of these insect sperm to produce these unusual beating patterns, possibly, in part, by interactions with the accessory microtubules and associated proteins surrounding the axoneme.

We conducted a proteomic analysis of mature *Culex pipiens* sperm as a first step in identifying and characterizing the major regulatory components of the flagellum and for comparison to proteomes of sperm from other species. This analysis identified a number of kinases, phosphatases, and Ca^2+^ regulators that may be important in controlling flagellar waveform, including an Erk1/2 MAPK, which controls switching between flagellar waveforms, as shown by pharmacological treatments in our earlier studies [5].

In addition, we detected a protein, previously annotated only as a conserved hypothetical protein, that bears homology to an axonemal dynein heavy chain (DHC10) and we hypothesize that the protein may reside on the accessory microtubules of the axoneme and contribute to waveform patterns. Furthermore, the *Culex* sperm proteome contains an unusual β-tubulin isoform with a predicted mass of 70 kDa due to a C-terminal extension of nearly 200 amino acids. No other tubulins in the non-redundant protein databases with such a feature have been found, and the localization of this 70 kDa tubulin will be important for ultimately determining its function.

Here we summarize these and other important features of the Culex sperm proteome.

## Materials and methods

### Animals

#### Mosquito colony

*Culex pipiens* males were obtained 5–7 days post eclosion from a colony maintained by our colleague Dr. Edward Platzer (Departments of Nematology and Evolution, Ecology and Organismal Biology, University of California, Riverside). The colony is an autogenous, stenogamous population isolated in Dixon, CA and maintained in the lab since 1973 [6]. Animals were anaesthetized using CO_2_ and 3–5 males placed in small Solo® cups. Dilute sugar water was added to each cup to humidify the chamber and provide nutrients until animals were dissected to collect tissues.

#### Dissections

Animals were euthanized by placing a small piece of chloroform soaked cotton in the Solo® cup. The male reproductive tracts were dissected from the animals. For mass spectroscopy, the testes and external genitalia were removed, leaving the paired seminal vesicles and accessory glands. The accessory glands were dissected away from the seminal vesicles. The seminal vesicles were transferred to a droplet of PBS and forceps were used to squeeze sperm out of each seminal vesicle into the buffer. The seminal vesicle tissue was then removed. After isolating sperm from several animals (~30), the sample was transferred to a 0.65 mL microfuge tube and stored frozen (-20°C). Additional aliquots of sperm were collected and added to the microfuge tube until sufficient sample was collected for processing for mass spectroscopy. For isolation of genomic DNA, or testis RNA, tissue processing is described below.

### Proteomic analysis

#### Mass spectroscopy

We examined three sperm preparations by mass spectroscopy to compile the *Culex* sperm proteome. Our initial preparation was a cell suspension containing whole sperm from 75 males. The data analysis of this sample positively identified a relatively small number of proteins (523), and we therefore collected a larger sample, containing sperm from 300 males. This sample yielded slightly more identifications (611). Given that the number of unique IDs was substantially lower than for other published sperm proteomes [7–11], we prepared a third sample of sperm from 80 males using a pre-fractionation approach, reducing SDS-PAGE using a 4–15% gradient gel, in order to identify more, and possibly lower abundance, proteins. The gel lane was excised and cut into 6 pieces, with regions containing high abundance protein bands separated from regions containing low abundance protein bands. Each of the 6 gel pieces was prepared for MS and analyzed separately. This analysis enabled identification of significantly more proteins (1667 unique IDs) as each sub-sample contained fewer protein species initially, due to the SDS-PAGE gel fractionation.

Sample preparation and mass spectroscopy was performed by the Biomolecular and Proteomics Mass Spectrometry Facility at UC San Diego. Detailed protocols for preparation of cell suspensions and of gel pieces are available at https://bpmsf.ucsd.edu/training-protocols/protocols.html. Briefly, *Culex* sperm cell suspensions were treated with DTT in acetonitrile/ammonium bicarbonate buffer to reduce proteins, heat denatured at 95 C for 10 min, cooled, and trypsinized for 12 h. The reaction was quenched with trifluoracetic acid. The gel lane containing *Culex* sperm proteins was cut into six pieces, and each piece processed separately. After destaining, gel pieces were dehydrated in a speed vac and proteins reduced using DTT in ammonium bicarbonate buffer, alkylated in iodoacetamide/ammonium bicarbonate, and then dehydrated in a speed vac. Proteins were trypsinized overnight, and supernatant then collected. Peptides remaining in the gel were extracted using a formic acid/acetonitrile solution and pooled with the first supernatant.

All samples were analyzed by MS/MS using a TripleTOF 5600+ QTOF instrument (SCIEX, Framingham, MA).

#### Proteome assembly

Protein identifications were assigned from the mass spectrometry results using the MASCOT software (Matrix Science, London, UK).

Because the sperm isolation method involves squeezing the seminal vesicles to release sperm into the buffer, some proteins from the seminal vesicle wall may have contaminated the sperm samples. Since contaminating proteins would be expected to be in very low abundance compared to sperm components, we used the following rationale for inclusion or rejection of proteins identified by MS. To be included in the sperm proteome, each protein had to be represented by two or more peptide matches at >95% confidence across the three proteome replicates. These strict criteria filtered out weak identifications and allowed us to include only proteins with robust identifications as components of the *Culex pipiens* sperm proteome.

**Fig 1:**
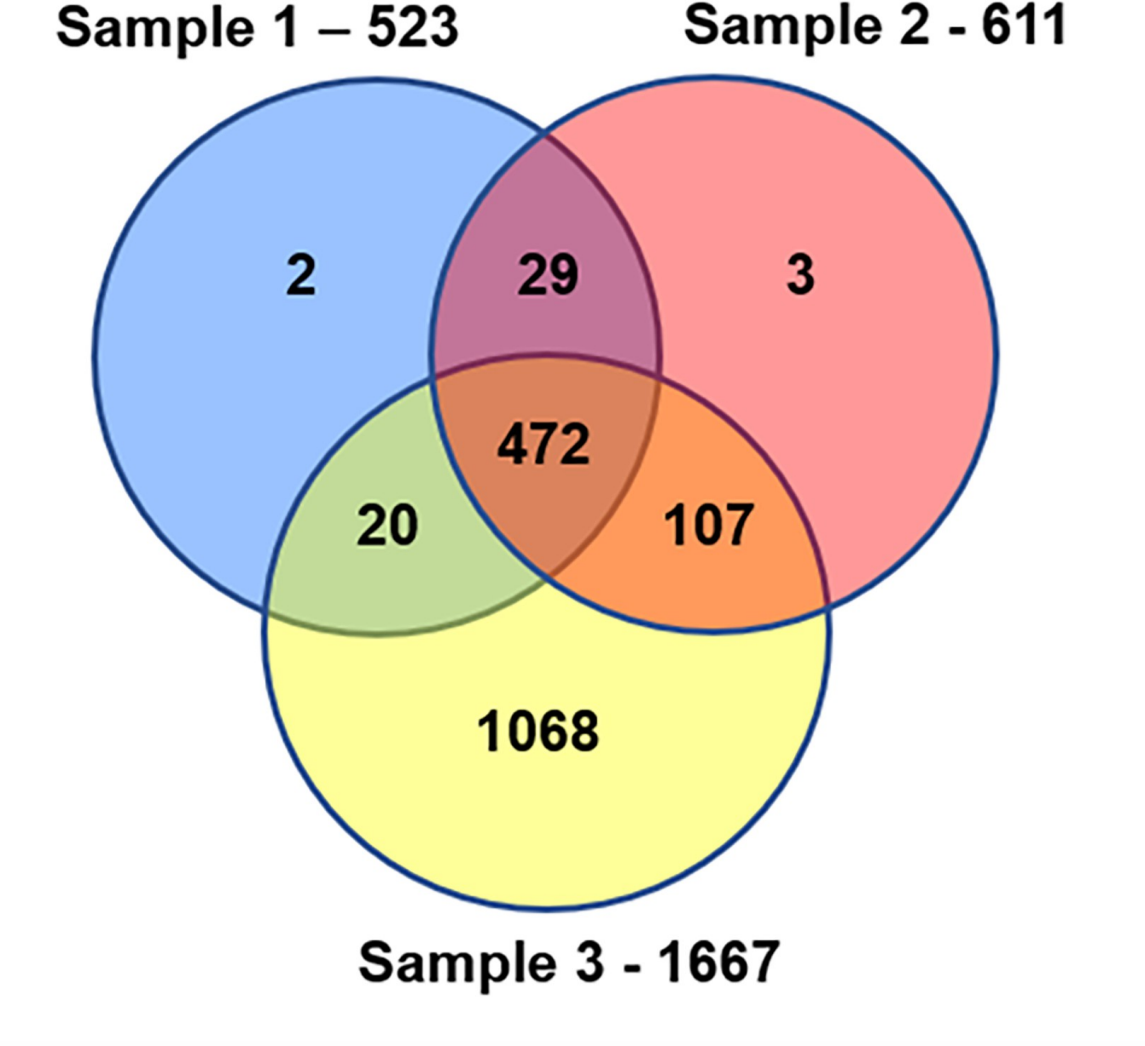
Distribution of unique protein identifications across three *Culex* sperm proteome samples.

The distribution of unique IDs across the three replicates is shown in Fig 1. The data for each unique ID is shown in the S1 Table, where the detection of each protein and the number of >95% confidence peptides detected in each of the 3 proteome samples is listed to the right of each entry.

The total number of unique IDs in each proteomic analysis is shown outside the corresponding circle. Overlaps show the proteins detected in more than one sample. Because the third proteomic sample was divided into sub-samples after gel fractionation of sperm proteins, the greatest number of protein identifications are derived from this sample.

We used the Blast2GO software within OMICS-Box (BioBam Bioinformatics, Cambridge, MA) for gene ontology analysis of the unique IDs and to generate charts showing protein distribution by biological process, molecular function, and cellular component.

#### DNA and RNA isolation and cDNA production

DNA isolation was performed on 10 whole *Culex* male bodies, excluding legs, wings, and heads. The bodies were placed in 500 μL Trizol™ (Invitrogen, Carlsbad, CA) and DNA isolated using the manufacturer’s protocol. RNA isolation was performed on no less than 100 *Culex* testes that were homogenized in Trizol™ reagent. RNA was extracted using the Zymo Direct-zol RNA Miniprep Plus (Zymo Research, Irvine, CA, catalog #R2073) according to the manufacturer’s protocol. Concentrations of both DNA and RNA were determined using a QUBIT® 2.0 Fluorometer (Invitrogen, Carlsbad, CA).

Aliquots of 100 ng testis RNA were converted to cDNA using the Zymo-Seq RiboFree Universal cDNA Kit (Zymo Research, Irvine, CA, catalog #R3001) using the temperature and cycle times provided in the manufacturer’s protocol.

#### PCR analysis of the 70 kDa tubulin and Erk1/2

Primers were designed using the NCBI-Primer BLAST software. Primers were obtained from Integrated DNA Technologies (Coralville, Iowa) and 10 μM Working Solutions were diluted from a 100 μM stock. Standard PCR reactions of 50 μL, using 25 μL MyTaq HS Red (Bioline, Memphis, TN), 2 μL of each primer, and 100 ng gDNA or cDNA, were conducted using the following conditions: denaturation at 95°C for 1 min, followed by 35 cycles of 95°C for 15 sec, 55°C for 15 sec, 72°C for 10 sec, and a final extension at 72°C for 5 min. PCR products were separated on 1.5% agarose gels in 1x TBE using a Bio-Rad horizontal gel rig at 130V for 30 min. PCR products were visualized by ethidium bromide staining of gels. Color images of gels were converted to grayscale for presentation in the figures. For Erk1/2, relevant lanes (taken from a single gel) were juxtaposed in a single image.

The relevant gene sequences, primer sequences, and predicted product sizes for this study are presented in the figures showing the PCR results.

#### Western blot analysis of Erk1/2

Sperm from the seminal vesicles of 200 male *Culex* were used for Western blot detection of ERK1/2. Mosquitoes were dissected as described above and isolated seminal vesicles were rinsed in a fresh aliquot of PBS to remove cells or other tissue. The seminal vesicles were then squeezed into an aliquot of fresh PBS in a deep-well slide. The well was sealed with parafilm and kept over ice to reduce evaporation and protein degradation during dissections. The sperm suspension was collected and transferred to a microcentrifuge tube. An equal volume of nanopure water was used to wash the collection well and collect any remaining sperm and was added to the sample yielding a 0.5x concentration of PBS. The samples were kept frozen at -20 C until sperm from all 200 seminal vesicles was collected. Sample volume was reduced using a Savant™ SpeedVac™ (ThermoFisher, Waltham, Massachusetts, USA.) vacuum concentrator with heat for 40–50 minutes.

Samples were resolubilized in a 1x reducing sample buffer for 30 minutes at 40 C. The sperm sample, an EGF-stimulated A431 cell lysate (Millipore, Temecula, CA) as a positive control, and pre-stained molecular weight markers (Bio-Rad, Hercules, CA) were boiled for 5 minutes. Samples were then frozen overnight and boiled again immediately prior to loading on the gel. The samples were separated on reducing 8% SDS-PAGE gels [12] at 60V until the dye front reached the bottom of the gel. Proteins were transferred to 0.22 μm nitrocellulose (Bio-Rad, Hercules, CA) using the method of Otter et al. [13] at 80V for 2 h. The membrane was stored at -20 C until used for Western Blotting.

The membrane was blocked in Tris buffered saline (TBS) containing 5% (w/v) non-fat dry milk and 0.1% Tween-20 (Blotto) for 30 min at RT. All subsequent incubations were conducted using Blotto at RT. The membrane was incubated in a 1:1000 dilution of a rabbit polyclonal ERK1/2 antibody (Invitrogen, Camarillo, CA, #44-654G) for 1.5 h. The membrane was washed 8 times for 5 min each to remove non-specifically bound antibody and then incubated in a 1:5000 dilution of an HRP-conjugated goat-anti-rabbit IgG (Jackson ImmunoResearch, West Grove, PA) for 1 h. The membrane was washed 12 times for 5 min each, to reduce non-specific binding and then incubated with Pierce Pico PLUS substrate (Thermo Scientific, Rockford, IL) for 5 minutes. Finally, the blot was exposed to film (Amersham Hyperfilm, Thermo Fisher Scientific, Waltham, Massachusetts, USA) for various times and developed using an automatic film developer (Mini-Med 90, AFP Manufacturing, Peachtree City, GA). The figure shows a 10 second exposure of the blot.

## Results & discussion

### Sperm proteome features

Our analysis produced 1,700 unique identifications of *Culex* sperm proteins, as well as ~100 ambiguous identifications among histones and 94 ambiguous identifications involving pairs or trios of highly homologous protein isoforms. The number of *Culex* sperm proteins identified is similar to the numbers of proteins that have been identified in sperm proteomes from other organisms including Drosophila 1,108 [7], macaque 1,247 [8], rat 829 [9], mouse 858 [10], human 1,056 [11]. The Chlamydomonas flagellum proteome [14] also contains a similar number of proteins, 1,134.

The accession numbers and annotations of the uniquely identified proteins are shown in the S1 Table. The non-histone ambiguous IDs are shown in the S2 Table and the histone ambiguous IDs are shown in the S3 Table. For the Tables presented in the main text, “*Culex pipiens*” has been removed from the annotations and only the categorical names of the proteins have been included.

**Fig 2:**
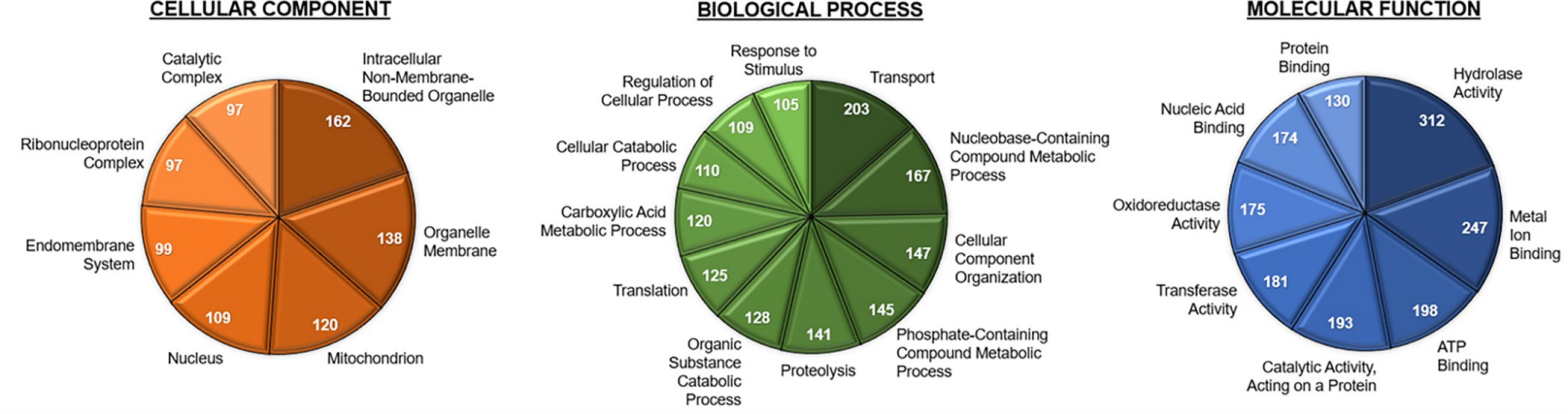
Protein distribution among unique IDs by gene ontology analysis. A) Cellular component. B) Biological process. C) Molecular function.

A gene ontology analysis of the unique IDs was conducted to describe the distribution of functional types of proteins found in the Culex sperm (Fig 2).

The S1 Fig. shows finer detail in gene ontology assignments and the accompanying S4 Table lists each gene assignment to each category, as determined by the Blast2GO software (BioBam Bioinformatics, Cambridge, MA).

Of the unique protein IDs in the *Culex* sperm proteome, 335 sequences (19.7% of the unique IDs) are annotated only as conserved hypothetical proteins, predicted proteins, or uncharacterized proteins. (In the J2 genome build, most of these have been renamed “uncharacterized” proteins). We used the NCBI conserved domain database [15] to identify known domain features of these proteins and this information is shown in the S5 Table. A subset of these proteins possessed domains that suggest they are axonemal components such as A-kinase-anchoring protein (AKAP), radial spoke head, and dynein intermediate or light chain domains. One polypeptide, discussed in more detail below, has several features suggesting that it is an axonemal dynein heavy chain. Some of these proteins (34, or 10.1% of the uncharacterized proteins) possessed highly conserved “domain of unknown function” (DUF) sequences, including one DUF (DUF1074) previously characterized as specific to *Drosophila melanogaster* [15], and a sizeable fraction (50 proteins, 14.9% of the uncharacterized proteins) had no known conserved domains. Given the large number of these uncharacterized proteins, it is likely that there is still a great deal about the regulatory and structural features of the insect sperm flagellum that is unknown.

In the following sections, we highlight key subsets of sperm proteins that are of particular interest in understanding sperm physiology, especially those involved in flagellar motility and motility regulation.

### Kinases and phosphatases

The kinases and phosphatases identified in the *Culex* sperm proteome are listed in Tables 1 and 2, respectively. These enzymes play key roles in signal transduction cascades mediating cellular responses and our preliminary functional analysis of *Culex* sperm motility indicated that kinase and phosphatase activity plays a key role in activating flagellar motility and in modulating flagellar waveform [5].

**Table 1:** Non-metabolic kinases and associated regulatory subunits detected in the *Culex* sperm proteome.

Gene ID	Annotation
CPIJ018980	calcium/calmodulin-dependent protein kinase type 1 (201 aa)
CPIJ018257	cAMP-dependent protein kinase catalytic subunit (354 aa)
CPIJ017648	calcium/calmodulin-dependent protein kinase type II alpha chain (491 aa)
CPIJ015942	c-AMP dependent protein kinase type I-beta regulatory subunit (343 aa)
CPIJ015672	inhibitor of nuclear factor kappa B kinase beta subunit (804 aa)
CPIJ015177	serine/threonine-protein kinase OSR1 (529 aa)
CPIJ013234	activated protein kinase C receptor (312 aa)
CPIJ011540	casein kinase II subunit alpha (343 aa)
CPIJ011538	rho-associated protein kinase 1 (1399 aa)
CPIJ010611	dual specificity mitogen-activated protein kinase kinase 4 MAPKK4 (389 aa)
CPIJ009957	serine/threonine-protein kinase SRPK2 (652 aa)
CPIJ009705	S-phase kinase-associated protein 1A (163 aa)
CPIJ008898	casein kinase (460 aa)
CPIJ008013	cyclin G-associated kinase (1195 aa)
CPIJ007754	rac serine/threonine kinase (545 aa)
CPIJ007354	testis-specific serine/threonine-protein kinase 6 (398 aa)
CPIJ005276	cGMP-protein kinase (109 aa)
CPIJ002636	kinase (417 aa)
CPIJ002609	serine/threonine kinase (127 aa)
CPIJ002175	mitogen-activated protein kinase 14 (122 aa)
CPIJ002174	mitogen-activated protein kinase 14B (208 aa)
CPIJ001596	testis-specific serine/threonine kinase (406 aa)
CPIJ001538	hepatocyte growth factor-regulated tyrosine kinase substrate (746 aa)
CPIJ001155	cell division protein kinase 2 (299 aa)
CPIJ000714	phosphatidylinositol 4-kinase (2153 aa)

**Table 2:** Phosphatases and associated regulatory subunits detected in the *Culex* sperm proteome.

GENE ID	Annotation
CPIJ018692	serine/threonine-protein phosphatase 2B catalytic subunit 2 (497 aa)
CPIJ016640	inositol monophosphatase (275 aa)
CPIJ015548	inositol polyphosphate 1-phosphatase (363 aa)
CPIJ012238	phosphatase 4 (985 aa)
CPIJ010401	phosphatase 1 regulatory subunit 7 (322 aa)
CPIJ010401	phosphatase 1 regulatory subunit 7 (322 aa)
CPIJ009620	serine/threonine-protein phosphatase 5 (507 aa)
CPIJ009405	testis/skeletal muscle dual specificity phosphatase (215 aa)
CPIJ008291	low molecular weight phosphotyrosine protein phosphatase 1 (162 aa)
CPIJ008212	phosphatase-1 (328 aa)
CPIJ008163	skeletal muscle/kidney enriched inositol 5-phosphatase (565 aa)
CPIJ003655	pap-inositol-1,4-phosphatase (312 aa)
CPIJ003532	serine/threonine protein phosphatase 2a regulatory subunit a (537 aa)
CPIJ001987	phosphatase-1 (348 aa)
CPIJ001969	tyrosine phosphatase prl (114 aa)
CPIJ001085	low molecular weight protein-tyrosine-phosphatase (153 aa)

#### Kinases

Several MAP kinases and testis specific kinases (TSSKs) were among the enzymes identified in the sperm proteome (Table 1). An ERK 1/2 MAPK (CPJ005303) which is of particular interest as a regulator of waveform transitions as suggested by earlier work [5], was detected by 1 peptide match only. Given the weak ID, further verification of its presence in mature sperm was warranted (see the MAPK section below). MAPK14 and MAPK14B are p38-like MAPKs that are present in *Culex* sperm. Both ERK 1/2 and p38 kinases have been implicated as regulators of human sperm motility [16].

The testis specific serine/threonine kinases (TSSKs) were first identified as novel kinases expressed specifically in mouse testis [17, 18] that played a role in spermiogenesis. They have since been identified in many animal taxa, both invertebrate (scallop [19]) and abalone [20]) and vertebrate (mouse [18, 19], bull [21], human [22]). However, subsequent work has shown that several TSSKs are present in mature mouse and human sperm [22], suggesting that they may play a role in the physiology of mature sperm, in addition to their roles in sperm morphogenesis. A TSSK6 (CPIJ007354) and a TSSK4-like protein (CPIJ001596) were found in *Culex* sperm. Although the presence and subcellular localization of TSSKs in mature mouse and human sperm [22] has raised intriguing questions, the function of TSSKs in mature sperm has not been previously reported in any system, and *Culex* may be amenable to further study to localize and examine the role of these regulators in sperm function.

Kinases related primarily to metabolic processes (e.g., glycolysis, tricarboxylic acid cycle, electron transport, etc.) are not included in Table 1, but can be found in the complete listing of unique IDs.

#### MAPK

In our previous work investigating the regulation of flagellar waveforms, we identified MAPK as a key regulator of those waveforms [5]. Our pharmacological analysis showed that inhibition of MAPK activity prevented virtually all sperm from switching from one waveform to another. These analyses used drugs specific for an ERK1/2 family member (U0126 which targets MEK, and FR180204 with targets Erk1/2), and immunofluorescence staining with an antibody that detects phosphorylation by MAPK and other proline-directed kinases showed phosphorylated substrates in the flagellum of activated *Culex* sperm but not in sperm fixed prior to motility activation [5].

**Fig 3:**
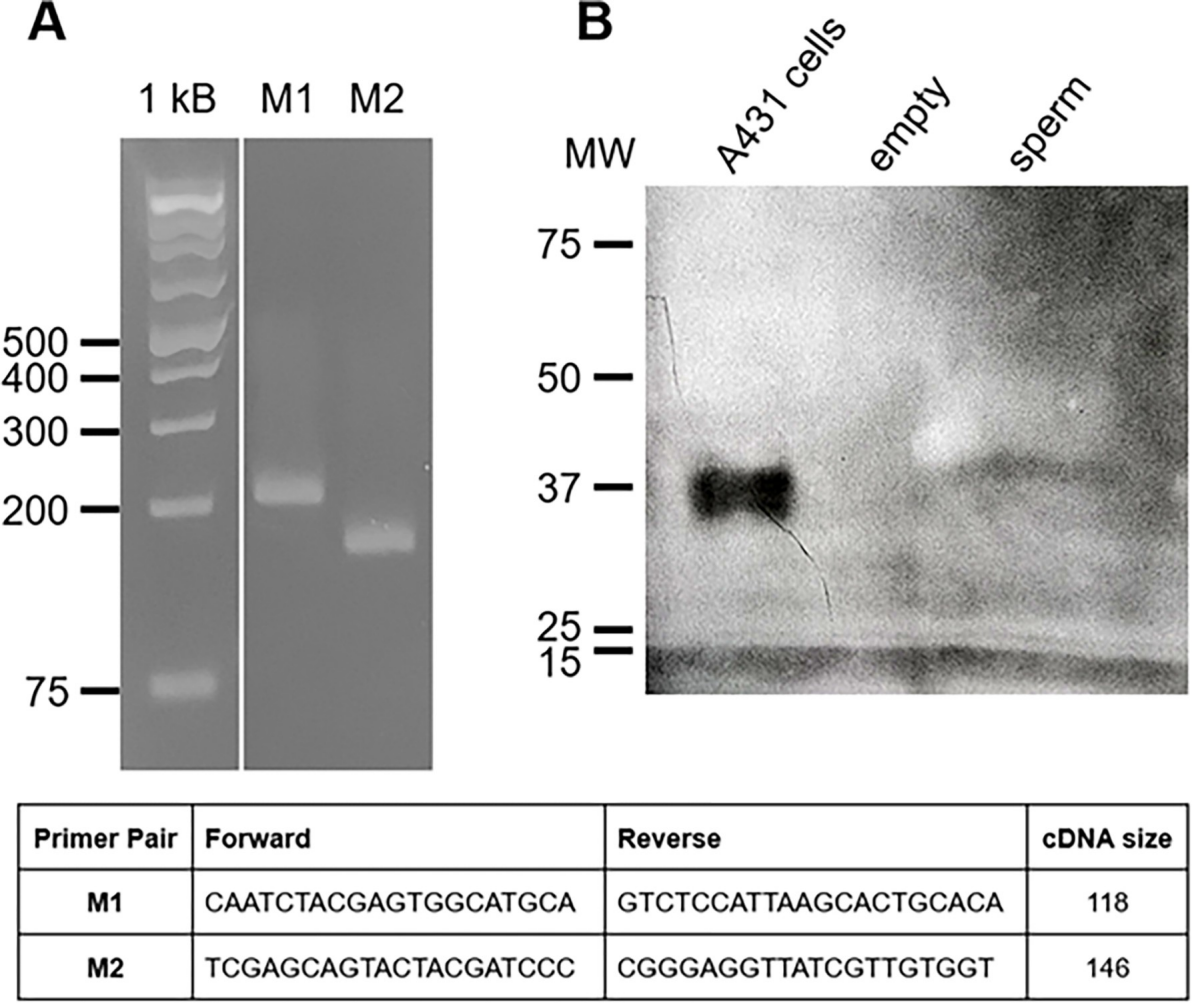
PCR and Western blot identification of MAPK. **A)** PCR analysis of testis cDNA for MAP Kinase. The primers used and predicted amplicon sizes are shown below the gel. **B)** Erk1/2 Western blot of *Culex* sperm. Using a polyclonal anti-Erk antibody, a band was detected in *Culex* tissues at approximately 42 kDa, consistent with migration of the positive control band from an EGF-stimulated A431 cell lysate.

In our proteomic analysis, we detected an ERK 1/2 MAPK (CPIJ005303). However, only 1 high confidence peptide was found. PCR of *Culex* testis cDNA confirmed that a transcript for ERK1/2 is expressed in the testis (Fig 3A). Erk1/2 was also detected by Western blotting of Culex male reproductive tract using an anti-Erk antibody (Fig 3B). Together with our earlier studies, these results suggest that an Erk1/2 MAPK is a component of *Culex* sperm.

#### Phosphatases

Phosphatases (Table 2) may have received less attention than kinases for their role in flagellar motility but are no less important in regulating overall levels of phosphorylation of target proteins (that control motility directly), as well as modulating kinase activity itself. In *Culex* sperm, we have shown that inhibition of phosphatases with okadaic acid is sufficient to initiate backward motility of sperm [5]. In *Culex*, we hypothesize that phosphatases play important roles in activating waveform changes in forward motility by controlling levels of MAPK activity.

Two isoforms of PP1 (CPIJ008212 and CPIJ001987), as well as PP2B (calcineurin) and several other phosphatases are present in the sperm proteome. A PP2B defect was recently shown to cause infertility in mouse due to changes in sperm motility [23]. One dual specificity phosphatase (CPIJ009405) that could be a negative regulator of MAPKK (MEK) [24, 25] was also found in the sperm proteome.

Thus, multiple potential regulators of the MAPK pathway that we have previously shown to play a key role in flagellar motility [5] are present in *Culex* sperm and await further characterization of their role in motility.

### Ca^2+^ binding proteins

Calcium signaling plays important roles in sperm physiology, including initiating the acrosome reaction in mammalian sperm [26], regulating chemotaxis in echinoderm sperm [27, 28], and controlling initiation [29] and the direction [5] of sperm motility in insect sperm. A number of Ca^2+^ binding proteins were present in the *Culex* sperm proteome (Table 3). Several have been detected in other sperm, including calreticulin, calcyphosin, and calmodulin (CaM). Four proteins with EF hand motifs were identified in addition to the four CaM isoforms. Four proteins with C-type (Ca^2+^ dependent) lectin carbohydrate recognition domains (CRD) were also identified. Three Ca-ATPases were detected: two SERCA isoforms and a plasma membrane Ca-ATPase (PMCA).

**Table 3:** Ca^2+^ binding proteins detected in the *Culex* sperm proteome.

GENE ID	Annotation	Comment
CPIJ018434	calcium-transporting ATPase sarcoplasmic/endoplasmic reticulum type (815 aa)	
CPIJ018366	calmodulin (150 aa)	ambiguous w/ CPIJ007602
CPIJ018021	calcium-transporting atpase sarcoplasmic/endoplasmic reticulum type (996 aa)	
CPIJ017628	EF-hand domain-containing family member C2 (569 aa)	
CPIJ017380	calmodulin (150 aa)	
CPIJ016689	hypothetical protein (151 aa)	CLECT CRD domain
CPIJ015374	EF-hand domain-containing protein 1 (651 aa)	
CPIJ014869	conserved hypothetical protein (273 aa)	CaM binding
CPIJ013850	conserved hypothetical protein (154 aa)	TPD52 (Ca^2+^-mediated signal transduction)
CPIJ011379	calmodulin (159 aa)	
CPIJ009755	serrate protein (3711 aa)	
CPIJ009655	conserved hypothetical protein (463 aa)	EF1 domains
CPIJ009593	conserved hypothetical protein (370 aa)	CaM binding
CPIJ009505	conserved hypothetical protein (776 aa)	Na^+^/Ca^2+^ exchanger
CPIJ007602	calmodulin (168 aa)	ambiguous w/ CPIJ18366
CPIJ007376	calreticulin (410 aa)	
CPIJ006679	EF-hand calcium-binding domain-containing protein 1 (221 aa)	
CPIJ005984	conserved hypothetical protein (237 aa)	CLECT CRD domain
CPIJ005975	conserved hypothetical protein (186 aa)	EF Hand domain
CPIJ005963	sodium/calcium exchanger (597 aa)	
CPIJ005742	plasma membrane calcium-transporting ATPase 2 (1196 aa)	
CPIJ004607	conserved hypothetical protein (218 aa)	CLECT CRD domain
CPIJ002304	ef-hand protein nucb1 (579 aa)	
CPIJ002079	C-type Lectin (142 aa)	CLECT CRD domain
CPIJ001560	calcium-binding protein (192 aa)	
CPIJ000250	conserved hypothetical protein (1141 aa)	EGF_C (Ca^2+^ binding) domain
CPIJ000193	calcyphosin (216 aa)	

SERCA has been identified in mammalian sperm (human, mouse, and bull) where its localization over the acrosome [30] and the sensitivity of acrosomal exocytosis to drugs such as thapsigargin [31] have implicated SERCA in acrosomal exocytosis. In contrast, other studies implicate the secretory pathway Ca^2+^ ATPase (SPCA) in Ca^2+^ signaling in human sperm [32]. Many insects possess an acrosome at the anterior of the sperm head, but studies to date suggest that insect sperm do not undergo an acrosome reaction [33]. Thus, Ca^2+^ regulators such as the SERCA isoforms found in *Culex* sperm may be more likely to mediate changes in intracellular Ca^2+^ required for control and modulation of flagellar waveforms.

In addition to the acrosome, the redundant nuclear envelope, in the sperm neck [34] is a potential internal Ca^2+^ store in mammalian sperm. Calreticulin localizes to this store and has been implicated in hyperactivated motility in mouse sperm [35]. Calreticulin is present in *Culex* sperm, and its subcellular localization may provide clues as to its potential function in sperm. *Culex* [2] and other insect sperm [36] do retain internal membranes in the flagellum suggesting the possibility that internal Ca^2+^ stores and regulators such as SERCA and SPCA, as well as Ca^2+^ binding proteins such as calreticulin, could modulate flagellar motility.

Another important regulator of sperm physiology in a number of species, the plasma membrane Ca^2+^ ATPase (PMCA), is also a component of *Culex* sperm. PMCA4 plays a role in hyperactivated motility of mouse sperm [37] potentially by coordinating nitric oxide (NO) and Ca^2+^ signaling [38]. A PCMA has been implicated as a sensor for sperm chemotaxis in an invertebrate model system [39]. PCMA has also been proposed as a target for contraceptives [40]. Data from vertebrate models suggest that the PCMA4 isoform is specifically expressed in sperm, but reports from studies in the invertebrate models show a diversity of PCMA isoforms, depending on the taxa [39, 41]. The *Culex* sperm PCMA is homologous to PCMA2 and its role in Ca^2+^ dependent motility behaviors will be important to evaluate in future studies.

Calcyphosin is an EF-hand containing Ca^2+^ binding protein first identified in dog thyroid [42], and later in many species including human [43–45]. A calcyphosin-2-like protein has been detected in human sperm [46], but its role in sperm physiology is unknown.

Several CaM isoforms were detected in *Culex* sperm; the isoforms encoded by CPIJ017380 and CPIJ011379 are definitively identified. One or both of the CPIJ018366 and CPIJ007602 isoforms are also present. CaM isoforms can play different roles in sperm physiology based not only on subcellular localization and interactions with other Ca^2+^ binding proteins such as the SERCA and PCMA, but also on differing Ca^2+^ sensitivity of the isoforms. The high degree of cooperativity in Ca^2+^ binding to the four EF-hand motifs means that minor differences in sequence can influence Ca^2+^ binding affinity and lead to activation of different isoforms under different [Ca^2+^]i and thereby enable differential regulation of flagellar machinery [47].

### Arginine kinases (CPIJ002029 and CPIJ007538)

Two arginine kinase gene products were detected in high abundance in the *Culex* sperm proteome. Arginine kinase (AK) is a guanidino kinase that uses the energy storage phosphagen arginine-phosphate (AP) as an ATP regenerating system similar to the creatine kinase/creatine-phosphate (CK/CP) system. Phosphagen/phosphagen kinase systems are widespread in the animal kingdom and are expressed in multiple tissues [48]. Some taxa have multiple phosphagen/phosphagen kinase systems, but arthropods express only AK/AP [48]. CK has been detected in sperm from many taxa, most notably Echinoderms, where Tombes and Shapiro [49, 50] showed that sea urchin sperm CK activity was necessary for sustained beating of the flagellum along its entire length. In sperm that depend on aerobic respiration for energy production and that have mitochondria located only at the base of the head, ATP generated by the mitochondria would take enormously long times to diffuse down the length of the flagellum [51]. Thus, CK/CP are proposed to form an energy shuttle system to maintain high levels of ATP throughout the flagellum.

Among arthropods, AK activity has been detected in moth, barnacle [50], and horseshoe crab [52] sperm. Although the role of AK/AP in sperm motility was not examined directly, the hypothesis is that AK/AP form an energy shuttle system similar to CK/CP in sea urchin sperm. In insect sperm, mitochondria are fused into two mitochondrial derivatives that typically run parallel to the axoneme down the length of the flagellum. However, it is not clear whether these structures retain the capacity for aerobic respiration since sperm motility in some insect species is completely unaffected by mitochondrial poisons [53]. In *Culex* spp. the mitochondrial derivatives contain a paracrystalline matrix [2], (and see aminopeptidase section below) that is suggested to form an elastic element of the flagellum and not be involved in respiration [54]. If the mitochondrial derivatives are not used in respiration, the sperm would depend on stored ATP, glycolysis, and/or an AK/AP regenerating system to supply ATP to power flagellar motility. However, a number of enzymes from the glycolytic and tricarboxcylic acid cycle pathways are present in *Culex* sperm. Thus, whether the sperm AK/AP system constitutes the only energy pathway available in the sperm, or works in concert with glycolytic and/or aerobic respiration pathways, remains to be assessed.

### Cytosol aminopeptidases

Amino peptidases are metallopeptidases that modify the N-terminus of peptides, and, as such, were thought to be involved in cellular homeostasis [55]. Other studies, however, showed that aminopeptidases have roles in a range of processes as diverse as transcriptional repression and vesicle trafficking [55]. In *Drosophila melanogaster*, aminopeptidases expressed in sperm have been localized to the mitochondrial derivatives and may have a structural role in the flagellum [56]. One hypothesis is that the paracrystalline matrix forms an elastic element that modifies flagellar bending characteristics [54] and, thus, may be involved in generating the distinctive waveforms observed in insect sperm [1].

Three cytosol aminopeptidase isoforms were abundant in the *Culex* sperm proteome (CPIJ009640 (XM_001850894.1), CPIJ003539 (XM_001845127.1), and CPIJ000990 (XM_001842608.1). These three genes are orthologs of the highly abundant sperm leucine aminopeptidase (S-LAP) proteins found in *Drosophila melanogaster* sperm [57]. Dorus et al. [57] showed that CPIJ009640 (XM_001850894.1) and CPIJ000990 (XM_001842608.1) are S-LAP Cluster II orthologs, and CPIJ003539 (XM_001845127.1) is an S-LAP Cluster I ortholog.

In Drosophila sperm, the S-LAPs are present at approximately 2-fold higher abundance than the tubulins, and, thus, have been suggested to play a structural role in the sperm flagellum [57]. Indirectly supporting this hypothesis is the fact that several of the isoforms found in sperm have mutations resulting in loss of catalytic activity. Recently, the paracrystalline matrix of the sperm mitochondrial derivatives in *Drosophila melanogaster* was shown to be comprised of S-LAPs [56], consistent with the hypothesis that the mitochondrial derivatives form a structural component of the flagellum. The paracrystalline matrix has previously been suggested to be an elastic element involved in determining the waveform of the flagellum [54], but how the structure of S-LAPs in the paracrystalline matrix generates this biophysical characteristic remains to be determined.

In *Culex*, three additional leucine aminopeptidases (CPIJ016662, CPIJ009825, CPIJ002841), as well as a glutamyl aminopeptidase (CPIJ011103) and a methionine aminopeptidase (CPIJ005763), were found in the sperm proteome. Whether they have a similar structural role, or some other function, is unknown.

### Histones and protamines

Many possible histones were detected in the *Culex* sperm proteome (S3 Table). A histone H2A variant (CPIJ008494) and a histone H2B variant (CPIJ020276) were unique identifications. The other identifications were ambiguous due to high sequence homologies but several groups were separately identified: 1) a histone H4 and histone 1 group (24 possible genes), 2) a histone H2B group (9 genes) 3) a histone H3 group (27 genes) including several H3.3 variants, 4) a histone H2A group (20 genes), including several late histones, 5) a pair of histone H3 isoforms (2 genes), and 6) a group of histone H1 variants (17 genes).

Although the IDs are ambiguous due to the high degree of sequence similarity among histones, the striking feature of the proteome is that only histones were detected and no protamines were found. Therefore, we used the *Drosophila* protamine A and protamine B sequences as queries and blasted [58] against the *Culex* genome, to determine if any protamine sequences could be detected. We found one protein with modest sequence similarity to the Drosophila Protamine A, isoform B (E = 6e-10, Query coverage 45%, Percent Identity 45.6%) and Protamine B (E = 7e-09, Query coverage 42%, percent identity 44.2%)—the conserved hypothetical protein CpipJ_CPIJ004430. This protein was detected in all three proteome samples. Given the abundance of histone peptides (if not the specificity of histone isoforms), detected in the mature sperm samples, our data suggest that *Culex* use primarily histones in the condensed sperm nucleus. Other studies examining the evolution of sperm chromatin remodeling proteins has suggested that some taxa retain histones in sperm [59 - see Box 1 in text]. The protamine-like CPIJ004430 gene product is likely to also be localized to the condensed sperm chromatin, but understanding of its precise role in nuclear remodeling, if any, would depend on future experiments to verify that it has functional similarity to protamines in addition to the detected sequence similarity.

### Importins

Several nuclear trafficking proteins, including nine importins (importinα and importinβ family members) as well as ran, a ran-binding protein, and five exporter proteins, were detected in the *Culex* sperm proteome (Table 4). Movement of macromolecules across the nuclear envelope is tightly controlled in cells. Since the discovery of nuclear localization sequences and nuclear import machinery [60, 61], many factors comprising the import-export machinery of eukaryotes have been identified. Importins interact with specific protein cargoes and move them into the nucleus. Alpha and beta importins interact with distinct, and in some cases, partially overlapping, subsets of proteins [62, 63]. The array of importins expressed in a cell will therefore determine which cargoes enter the nucleus. Ultimately, access to the nucleus determines gene expression profiles and, thus, differentiation processes during spermiogenesis, as well as supplying the machinery that creates the condensed nucleus of the mature sperm.

**Table 4:** Nuclear import/export proteins detected in the *Culex* sperm proteome.

Gene ID	Name/Annotation
CPIJ017677	importin alpha-7 subunit (523 aa)
CPIJ012645	nuclear RNA export factor 2 (746 aa)
CPIJ011802	importin alpha re-exporter (974 aa)
CPIJ010329	importin beta-3 (1104 aa)
CPIJ009366	chromosome region maintenance protein 1/exportin (1054 aa)
CPIJ008595	ran (215 aa)
CPIJ008197	Importin9 (1018 aa)
CPIJ007834	RNA and export factor binding protein (291 aa)
CPIJ007518	ran-binding protein (2690 aa)
CPIJ004613	importin subunit beta (880 aa)
CPIJ004446	importin beta-3 (390 aa)
CPIJ002334	importin-4 (1081 aa)
CPIJ002096	importin-7 (1043 aa)
CPIJ001179	importin alpha (517 aa)
CPIJ000096	importin beta-2 (903 aa)
CPIJ000578	conserved hypothetical protein (1100 aa) (Importin-beta N-terminal domain; Exportin 1-like protein)

In mammalian systems, importins show temporally distinct expression profiles during spermatogenesis [64–66], suggesting that these proteins have specific roles mediating differentiation of the post-meiotic cell into a mature sperm. Interestingly, the sperm used in this proteomic study were all fully mature sperm collected from the seminal vesicles. As such, the importins and exportins detected may provide a “history” of import proteins used during mosquito spermiogenesis.

In addition to the role of importins in nuclear trafficking, recent studies have shown that some importins play roles in the cytoplasm [67, 68] which may include scaffolding, as well as response to oxidative stress [69]. Thus, it may be important to examine the role of these proteins in differentiation steps outside the nucleus during spermiogenesis.

### 70 kDA tubulin (CPIJ000407)

Microtubules are composed of α- and β-tubulin dimers. Most eukaryotes have several isoforms of each tubulin monomer in their genomes and, among the β-tubulins, testis and sperm specific isoforms have been identified in *Drosophila melanogaster* [70, 71]. Tubulins are a highly conserved gene family encoding proteins that have a molecular mass of approximately 55 kDa. The tubulin core is globular with approximately 15 amino acids at the C-terminus forming a disordered “tail” that protrudes from the globular region and is exposed on the surface of the microtubule [72]. The tails are subject to many post-translational modifications that are proposed to affect microtubule stability [72, 73], as well as interactions with microtubule binding proteins [72, 73]. The tails are important for polymerization of microtubules [72, 74] and are substrates for several enzymes that modify the tail by adding different moieties, including glutamate, glycine, and phosphate in the case of β-tubulins [72, 73, 75].

The β-tubulins are important for axoneme structure [76–79] and, thus, play a key role in fertility through their influence axoneme assembly and stability, and sperm motility. The β-tubulins also influence dynein motor function [80–82].

Five β-tubulin isoforms were detected in the *Culex* sperm proteome (Table 5), including a β-4-tubulin that has a C-terminal extension of nearly 200 amino acids (CPIJ000407). This *Culex* β-4-tubulin contains the highly conserved core region (93–98% amino acid identity to other insect β-tubulins, Thaler, BLAST search), as well as the less conserved tail sequence, and then has 190 amino acids at the C-terminus, giving a total predicted mass of 70 kDa (Prosite, https://prosite.expasy.org). The C-terminal extension has not been detected in any other tubulin in the non-redundant protein databases (Thaler, BLAST search).

**Table 5:** Tubulin isoforms detected in the *Culex* sperm proteome.

Gene ID	Annotation
CPIJ019940^a^	tubulin alpha-1 chain (451 aa)
CPIJ017383^a^	tubulin alpha-1 chain (451 aa)
CPIJ014155	tubulin alpha chain (439 aa)
CPIJ012634	tubulin beta-3 chain (457 aa)
CPIJ011550	tubulin alpha-2 chain (450 aa)
CPIJ010049	tubulin alpha chain (438 aa)
CPIJ006724	tubulin alpha-1 chain (451 aa)
CPIJ005487	tubulin gamma-1 chain (455 aa)
CPIJ003635	tubulin beta-1 chain (467 aa)
CPIJ003263	tubulin beta chain (450 aa)
CPIJ003260	tubulin beta chain (447 aa)
CPIJ001353	tubulin alpha chain (426 aa)
CPIJ000407	tubulin beta-4 chain (636 aa)

^a^These isoforms are ambiguous IDs: one or both may be present in sperm.

The uniqueness of this 70 kDa β-tubulin isoform leads to a number of questions, not the least of which being what is the role of this highly modified tubulin in sperm motility? Key features discussed below are highlighted in S2 Fig.

The core sequence of the 70 kDa tubulin contains several motifs that have been identified in other sperm tubulins as important determinants of sperm function. Raff et al. [83] identified an axonemal β-tubulin motif in the tubulin tail (EGEF followed by 3 acidic amino acids) that they found in diverse taxa in tubulins occurring in motile axonemes. Additionally, Nielsen, et al. (2001), showed that the motile axoneme motif of the Drosophila sperm β-2 tubulin specified the central pair microtubules and that mutations in the motile axoneme motif in Drosophila sperm perturbed central pair assembly [84]. The 70 kDa tubulin contains the motile axoneme motif (aa # 433–439), which may support the hypothesis that the 70 kDa tubulin will be found in the axoneme or in the accessory tubules surrounding the doublet microtubules of the insect sperm flagellum. The axoneme of *Culex* spp. is a 9+9+1 structure, with the central pair microtubules replaced by a single rod-like element that does not have the typical appearance of a microtubule [2–4]. The molecular composition of this central rod is unknown. One possibility is that the central rod is composed of tubulin dimers using the 70 kDa β-tubulin, giving the structure its unusual appearance.

An internal variable region (IVR) involved in outer dynein arm (ODA) attachment has been identified in β-tubulins from *Drosophila melanogaster* sperm [85]. A glycine residue at aa56 is required for ODA binding [85], and changes in this amino acid result in axonemes lacking ODAs; binding of inner arms is not affected. The *Culex* 70 kDa tubulin contains a glycine residue at the homologous position (S2 Fig).

The mass spectrometry results indicate that this β-tubulin is an abundant sperm protein. Given the role of tubulin tails in stability and interactions with other proteins, we also examined the secondary structure of this β-tubulin using a secondary structure prediction algorithm [86]. The C-terminal extension is predicted to be almost entirely alpha helices interspersed with short random coil motifs. These data raise a question as to how proteins would interact with the tubulin tail, or if the C-terminal extension forms a new interaction domain for this tubulin.

The 70 kDa tubulin could be located in the 9 accessory microtubules and the IVR glycine or the C-terminal extension could act as a docking site for the large dynein-like structures located on the accessory microtubules [4]. Alternatively, the 70 kDa tubulin could be located at specific sites along the doublet microtubules or in the accessory microtubules and be involved in generating the unusual double waveform motility that we and others have reported [3, 5, 87–91].

Although the 70 kD tubulin contains the “motile axoneme” motif, a different hypothesis is that it localizes to the nucleus-associated microtubule manchette. Sperm from *Culex pipiens* and *Culex quinquefasciatus* are distinct among insects in that the nuclear manchette is maintained in mature sperm [5] (and Thaler, unpublished data), whereas, in most species of insects, the manchette is disassembled after nuclear condensation.

These alternative hypotheses can be addressed in future studies. However, due to 1) the unique nature of this β-tubulin sequence, and 2) the fact that the MS peptides identifying the 70 kDa tubulin are from sequences within the tubulin core and not from the C-terminal extension, it was also important to determine whether the published genomic sequence is correct and expressed in its entirety in the testis.

We used PCR primers spanning different regions of the 70kD tubulin sequence to analyze both the genomic DNA and testis cDNA. (See S2 Fig for the primer locations and predicted amplicons sizes.)

**Fig 4:**
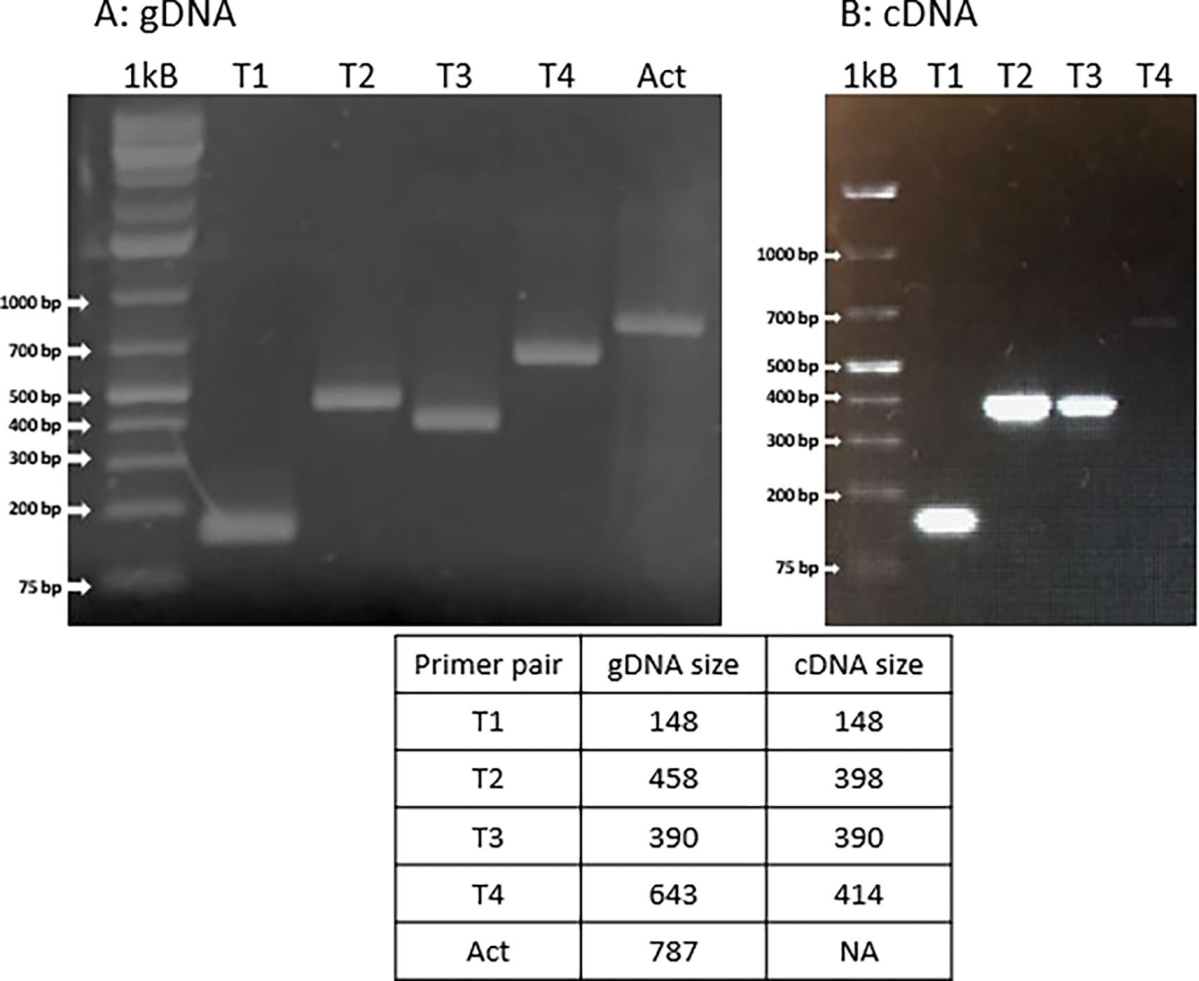
PCR amplicons from the 70 kD tubulin. **A: gDNA.** PCR products covering regions of the tubulin core (T1, T2, T3 primer pairs) as well as the C-terminal extension (T4 primer pair) were amplified from the genomic DNA. Primers targeting actin (Act) served as a positive control. The T4 amplicon includes core, tail, and C-terminal regions of the gene, thus indicating that the gene sequence does have the C-terminal extension. **B: cDNA.** The primers covering the tubulin core (T1, T2, T3) amplified sequences from the testis cDNA, but the T4 primers, covering the C-terminal extension did not produce a product from testis cDNA.

The gDNA results show that the genomic sequence is correct (Fig 4). The T4 primer set covers part of the tubulin core, just N-terminal to the variable tail region, across two small introns and into the coding sequence of the C-terminal extension (see S2 Fig). The T4 amplicon matches the predicted size for the gDNA sequence, thus indicating that the C-terminal extension is present as indicated in the published genome. However, the T4 primers did not amplify a product from the testis cDNA, thus we are not able to confirm that the C-terminal extension is expressed. Failure to amplify a product from the C-terminal extension could be due to a number of technical issues such as the stability of this particular mRNA or formation of hairpins or other secondary structures that interfere with amplification. Alternatively, the final transcript may indeed lack the C-terminal extension sequence. Future investigations may resolve this issue and set the stage for further investigations of the role of the 70 kDa tubulin in axonemal function.

### Novel dynein heavy chain (CPIJ001823)

This conserved hypothetical protein sequence codes for 5,022 amino acids and, in addition to its size, a conserved domain search [15] identified several domain features reminiscent of a dynein heavy chain (DHC), including N-terminal DHC regions 1 and 2, AAA ATPase domains, and the C-terminal D6 motor domain. A BLAST analysis indicated that the protein is most similar to an axonemal DHC10. Interestingly, 6 other annotated dynein heavy chains were detected in the *Culex* sperm proteome. Axonemal dyneins are well characterized proteins, so finding this gene product among the conserved hypothetical proteins was surprising.

TEM images of *Culex* sperm flagella show a typical appearance of inner and outer dynein arms associated with the 9 doublet microtubules. The novel DHC could form part of these dynein arms, or, alternatively, may be part of the dynein arm-like structures that sit on the 9 singlet accessory microtubules surrounding the doublet microtubules that attach to the adjacent doublets [2, 4].

Two other conserved hypothetical proteins (CPIJ011070 and CPIJ003818) have similarities to dynein heavy chain domains, but seem too small to form actual heavy chains. Their functions remain to be elucidated.

## Conclusions

Given that transcriptomics is not possible with mature sperm, the proteomic approach has been essential to identify sperm-associated proteins that are important in key physiological events that are essential for reproductive success. These include proteins involved in sperm maturation in both the male and female reproductive tracts, sperm storage, and the activation and maintenance of sperm motility that is essential for successful fertilization. The *Culex* sperm proteome provides a tool to facilitate targeted studies across a wide range of questions concerning gamete differentiation and function. Possible areas of future work have been discussed in reference to groups of proteins highlighted in this presentation and are only a few of the potential avenues made more accessible by the availability of this proteomic work.

Proteomes of sperm from a number of model species, mostly mammals, have been published [8–11]. The *Drosophila* sperm proteome represents an insect model proteome [7]. In insects, only a few studies have been presented for other species. Examinations of sperm from Lepidopteran species have focused on comparison of the proteomes of eupyrene and apyrene sperm [92–94]. Some studies have looked at *Apis mellifera* sperm to identify factors that may contribute to sperm longevity in the female tract [95]. A partial sperm proteome for *Aedes aegypti* has been published as part of a study focused on seminal fluid proteins transferred to the female upon copulation [96]; a similar study examining proteins transferred in semen in crickets also identified sperm proteins [97].

The *Culex* sperm proteome presented here is thus one of a growing number of studies examining insect reproductive biology, and as such will be valuable for both comparative investigations within the Order as well as those between other taxa, including well-studied marine invertebrate and mammalian model systems. Of particular interest are investigations of flagellar motility, especially in those systems where the axoneme deviates from the canonical “9+2” structure that is conserved throughout much of the animal kingdom [2]. Substantial modifications to these axonemal structures, especially the impressively diverse array of axonemes and their protein constituents in insect sperm [2], raises questions regarding differences in flagellar behavior in response to various environmental factors. Identification of those proteins is a necessary step in elucidating the function of those proteins and, as is the case for mosquitoes, potentially serve as molecular targets for population control.

## Supporting information

S1 Table List of unique IDs for *Culex* sperm proteome. The identifications are listed in order by gene ID and the annotations correspond to those published in the *Culex* quinquefasciatus genome (vectorbase.org). Columns B, C, and D show the number of peptides identified at >95% confidence for each protein ID in each of the three sperm samples analyzed by mass spectroscopy.
(XLS)

S2 Table List of histone IDs for *Culex* sperm proteome. Most IDs for the histones were ambiguous due to the high degree of sequence similarity among these genes. Sheet 1 of the file shows all of the possible histone IDs from the MS analysis. Sheet 2 shows the groups of ambiguous IDs (each ID group is separated from others by an empty row) found in the first two MS analyses. The third analysis of sperm proteins combined data from MS of proteins extracted from gel slices and are not included here, since they can not be considered comparable to the first two samples. The two histone isoforms that were uniquely identified are highlighted in pink.
(XLS)

S3 Table List of ambiguous IDs for *Culex* sperm proteome. Some protein IDs were ambiguous due to the high degree of sequence similarity among the isoforms. The pairs of ambiguous IDs are shown in Sheet 1 with an empty row between sets of ambiguous IDs. In a few cases, three or four proteins are potential candidates for the ID. Among these IDs, proteins of particular interest (e.g., calmodulin) are discussed in more detail in the Results and Discussion Section.
(XLS)

S4 Table List of gene ontology assignments for the unique IDs in the *Culex* sperm proteome. The table shows each gene ID counting in the number of sequences for each category. There is a separate sheet in the file for each analysis (i.e., Cellular Component, Biological Process, Molecular Function).
(XLSX)

S5 Table List of conserved hypothetical proteins in the *Culex* sperm proteome. The table lists all of the uncharacterized proteins that were detected in the sperm proteome. A short description of any conserved domains detected in each sequence is listed for each.
(XLS)

S1 Fig Gene ontology analysis of *Culex* sperm unique IDs. The figure shows detailed breakdown of gene ontology groups and the number of protein sequences assigned to each. Note that an individual protein can be included in more than one GO category. A) Cellular component. B) Biological process. C) Molecular function.
(DOCX)

S2 Fig Tubulin sequence. The nucleotide and amino acids sequences for the 70 kDa tubulin (CPIJ000407) are shown with highlighted features: a) regions corresponding to core, tail, and C-terminal extension, b) locations of conserved amino acids important in axonemes, c) PCR primer locations.
(DOCX)

S1 Raw images (PDF)
