# Supplementary figures and images for: Using the Culex pipiens sperm proteome to identify elements essential for mosquito reproduction
Source: PLoS One. 2023 Feb 16;18(2):e0280013. doi: 10.1371/journal.pone.0280013 (PMC9934393; doi:10.1371/journal.pone.0280013)

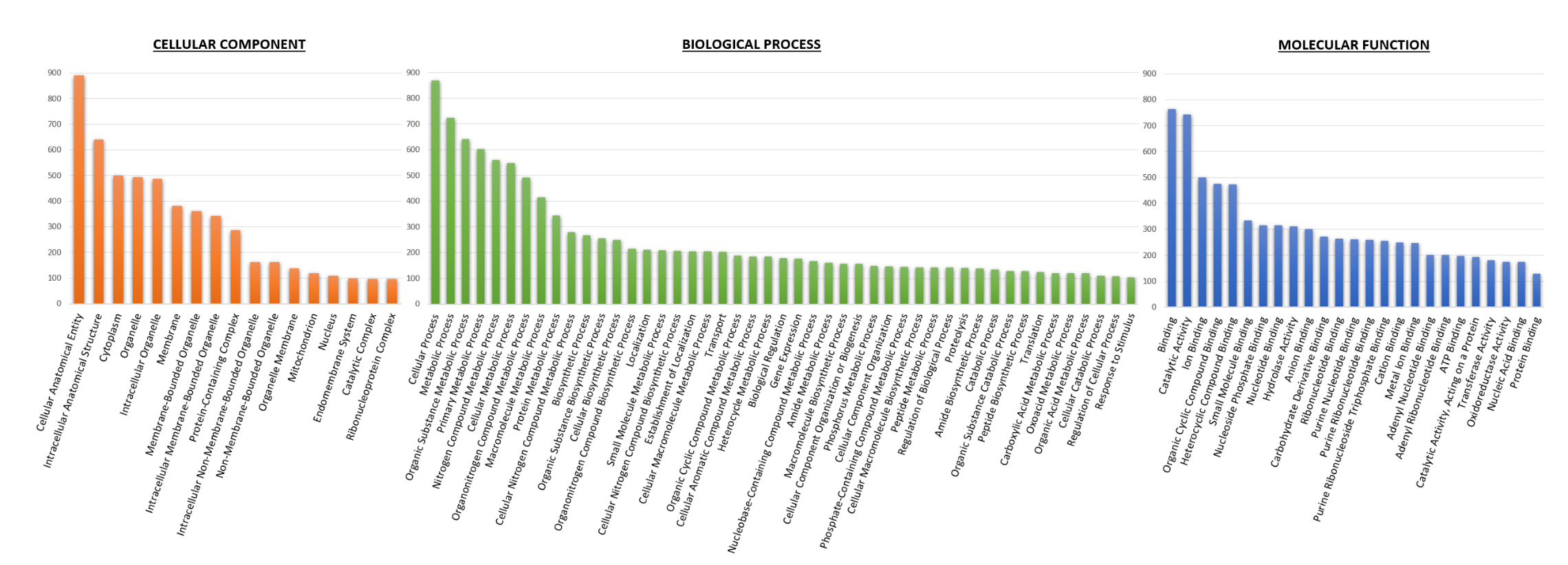

Supplement: S1 Fig — The figure shows detailed breakdown of gene ontology groups and the number of protein sequences assigned to each. Note that an individual protein can be included in more than one GO category. A) Cellular component. B) Biological process. C) Molecular function. (DOCX) [file pone.0280013.s006.docx]
