# Supplementary material for: Using the Culex pipiens sperm proteome to identify elements essential for mosquito reproduction
Source: PLoS One. 2023 Feb 16;18(2):e0280013. doi: 10.1371/journal.pone.0280013 (PMC9934393; doi:10.1371/journal.pone.0280013)

**SUPPLEMENTAL FIGURE 2.** SEQUENCE FEATURES OF THE 70kDa TUBULIN (CPIJ000407)

**1F** ATG CGT GAA ATT CTG CA**C ATT CAA GCG GGC CAG TG**C GGC AAC CAG ATC GGG GCG AAA TTT TGG GAG GTA ATT TCC

M R E I L H I Q A G Q C G N Q I G A K F W E V I S 25

**1R** GAC GAG CAC GGA ATC GAC GCG ACC GGA GCG TAC TGC GGC GAC AGC GAT CTG CAG CTG GAG CGG ATC AAC G**TG TAC**

D E H G I D A T G A Y C G D S D L Q L E R I N V Y 50

**TAC AAC GAG GCC ACC** GGC GGA AAG TAC GTT CCG CGG GCG ATC CTG GTG GAT CTG GAG CCG GGC ACG ATG GAC TCG

Y N E A T G G K Y V P R A I L V D L E P G T M D S 75

**2F** GTG CGA GCT GGC CCG TTT GGG CAG CTG TTC CGG CCG GAT **AAT TTT ATC TTT GGC CAG TCG G**GC GCC GGC AAC AAC

V R A G P F G Q L F R P D N F I F G Q S G A G N N 100

TGG GCC AAG GGA CAC TAC ACG GAG GGG GCC GAG CTG GTC GAT TCC GTG CTG GAC GTG GTC CGC AAA GAG GCC GAA

W A K G H Y T E G A E L V D S V L D V V R K E A E 125

GGC TGT GAC TGC ATG CAG GGC TTC CAG CTG ACG CAC TCG CTC GGG GGA GGA ACC GGT TCC **G-G**C ATG GGA ACG CTG

G C D C M Q G F Q L T H S L G G G T G S G M G T L 150

CTG ATC TCG AAA ATT CGC GAA GAG TAC CCC GAT CGC ATC ATG AAC ACG TTT TCG GTT GTT CCA TCG CCA AAG GTG

L I S K I R E E Y P D R I M N T F S V V P S P K V 175

TCC GAC ACG GTG GTT GAG CCG TAC AAC GCC ACG TTG AGC GTC CAC CAG CTG GTT GAG AAC ACG GAC GAA TCG TAC

S D T V V E P Y N A T L S V H Q L V E N T D E S Y 200

**2R** TGC ATC GAC AAC GAG GCC CTG TAC GAC ATC TGC TTC CGG ACG **CTA AAG TTG ACC ACG CCG AC**G TAC GGC GAT CTG

C I D N E A L Y D I C F R T L K L T T P T Y G D L 225

AAC CAC CTG GTG TCG GCG ACC ATG TCC GGC GTA ACG ACC TGT CTG CGC TTC CCC GGT CAG CTG AAT GCG GAT CTC

N H L V S A T M S G V T T C L R F P G Q L N A D L 250

**3F** CGC AAA TTG G**CC GTC AAC ATG GTT CCC TTT** CCG CGG CTG CAC TTT TTC ATG ACC GGC TTT GCA CCG CTA ACT TCG

R K L A V N M V P F P R L H F F M T G F A P L T S 275

CGA GGA TCT CAG CAG TAC CGT GCC CTG TCC GTC CCC GAG CTC ACC CAG CAA ATG TTT GAC GCG AAG AAC ATG ATG

R G S Q Q Y R A L S V P E L T Q Q M F D A K N M M 300

GCC GCG TGC GAT CCT CGA CAC GGA CGC TAC CTC ACG GTG GCC GCC ATC TTC CGA GGC CGG ATG TCC ATG AAG GAG

A A C D P R H G R Y L T V A A I F R G R M S M K E 325

GTG GAT GAG CAG ATG TTG AAC GTG CAG AGC AAA AAC TCC AGC TAC TTT GTC GAG TGG ATT CCG AAC AAC GTG AAG

V D E Q M L N V Q S K N S S Y F V E W I P N N V K 350

ACG GCC GTT TGC GAC ATT CCC CCT CGG GGT CTC AAG ATG TCC TCC ACC TTC ATC GGC AAC TCG ACT GCT ATC CAG

T A V C D I P P R G L K M S S T F I G N S T A I Q 375

**3R** GAG ATA **TTC AAG CGC ATC AAC GAA CA**G TTT ACG GCC ATG TTC CGG CGA AAG GCT TTC CTG CAC TGG TAC ACG GGC

E I F K R I N E Q F T A M F R R K A F L H W Y T G 400

**4F** GAG GGC ATG GAC GAG ATG GAG TTT ACC GAG GCC GAG AGC AAC **ATG AAC GAT CTG GTG TCC GA**G TAT CAA CAG TAC

E G M D E M E F T E A E S N M N D L V S E Y Q Q Y 425

CAG GAG GCT TCC GCG GAC GAG GAG GGA GAG TTC GAC GAG GAG GAG GAG GGC **G-A**T GTC CAC GAG CTG GCC GAG CAG

Q E A S A D E E G E F D E E E E G D V H E L A E Q 450

AAG CAG AAG CAA CTG CTC GAA AAG TAC ATT GCC CTG TAC CGG AAT CAC CGG TCG CTC CCT CGC ATC ACC GAC ACG

K Q K Q L L E K Y I A L Y R N H R S L P R I T D T 475

TAC CAC ATG GTG GCC AAC TTT TGC TTC CCG ACG CTG TTC GAG CCG CAG CTG AAG CGA CTC ATG GTC GTC CTG TAC

Y H M V A N F C F P T L F E P Q L K R L M V V L Y 500

CGG ACC GAT CCG GCC CAG TTT TGC GAG GTC ATC TCG CAG GCC GCC TTC CAG CTG CTG CTG TTT TAC AAG TCG GAA

R T D P A Q F C E V I S Q A A F Q L L L F Y K S E 525

**4R** TCG AGC GTC AA**G-A**AC CTG TTC AAA AAG TTC CAG GCG TTT GCC GGG GCC GAG CTG ACA GAG CAC G**CA GAC GAA AGT**

S S V K N L F K K F Q A F A G A E L T E H A D E S 550

**GCC AGC G**TG GTT TCA CGC GTC GTC GAG AAG ATT CTG AAC TAC ATG ATG GCG GCG GTG ATT GGA AAT GCG GAG AAT

A S V V S R V V E K I L N Y M M A A V I G N A E N 575

GCT CCC GGC GCC AAG AAG ATG CTC AAG CTG ATC GAA CCG ATG CTG CCG GCG ATG CCG GCT GAG GAG CTG GTC AAG

A P G A K K M L K L I E P M L P A M P A E E L V K 600

ATG GAG CAC CTG CTG GTG CGA CGG CAG GAG TAC GAA ACG CTG GAA CCG GCC GAG CGC AAG GTC ATC GAC AAG TTT

M E H L L V R R Q E Y E T L E P A E R K V I D K F 625

GTG CGG CAC TTG CGG AAG TGC GCG GAT GCG TAA

V R H L R K C A D A 635

The glycine essential for the ODA binding site (aa #56) is shown in purple highlight.

The tubulin tail sequence is shown with green and yellow highlights. The yellow highlights the motile axoneme motif.

Exon positions are indicated by red underline of the coding sequence nucleotides adjacent to the exon boundaries.

The PCR primer positions are shown with gray highlight and underline. The primer pair designations are shown in the left margin. Primer sequences and PCR product sizes for gDNA and cDNA are listed below. The primer pair for the actin positive control used in the gDNA reactions is also provided.

| Primer pair | Forward | Reverse | gDNA size | cDNA size |
| --- | --- | --- | --- | --- |
| T1 | CATTCAAGCGGGCCAGTG | GGTGGCCTCGTTGTAGTACA | 148 | 148 |
| T2 | AATTTTATCTTTGGCCAGTCGG | GTCGGCGTGGTCAACTTTAG | 458 | 398 |
| T3 | CCGTCAACATGGTTCCCTTT | TGTTCGTTGATGCGCTTGAA | 390 | 390 |
| T4 | ATGAACGATCTGGTGTCCGA | CGCTGGCACTTTCGTCTG | 643 | 414 |
| A1 | TTCAACTCGCCAGCCATGTA | TTTGTGGCGTTGTTTGGTTTG | 787 | NA |


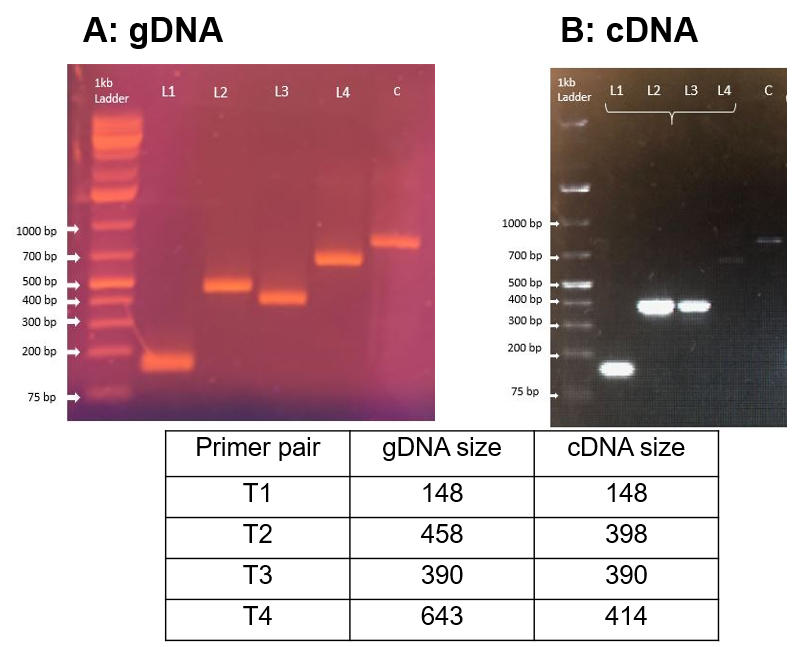

Supplement: S2 Fig — The nucleotide and amino acids sequences for the 70 kDa tubulin (CPIJ000407) are shown with highlighted features: a) regions corresponding to core, tail, and C-terminal extension, b) locations of conserved amino acids important in axonemes, c) PCR primer locations. (DOCX) [file pone.0280013.s007.docx]
