# Supplementary material for: Using the Culex pipiens sperm proteome to identify elements essential for mosquito reproduction
Source: PLoS One. 2023 Feb 16;18(2):e0280013. doi: 10.1371/journal.pone.0280013 (PMC9934393; doi:10.1371/journal.pone.0280013)
Supplement: S1 Raw images — (PDF) [file pone.0280013.s008.pdf]

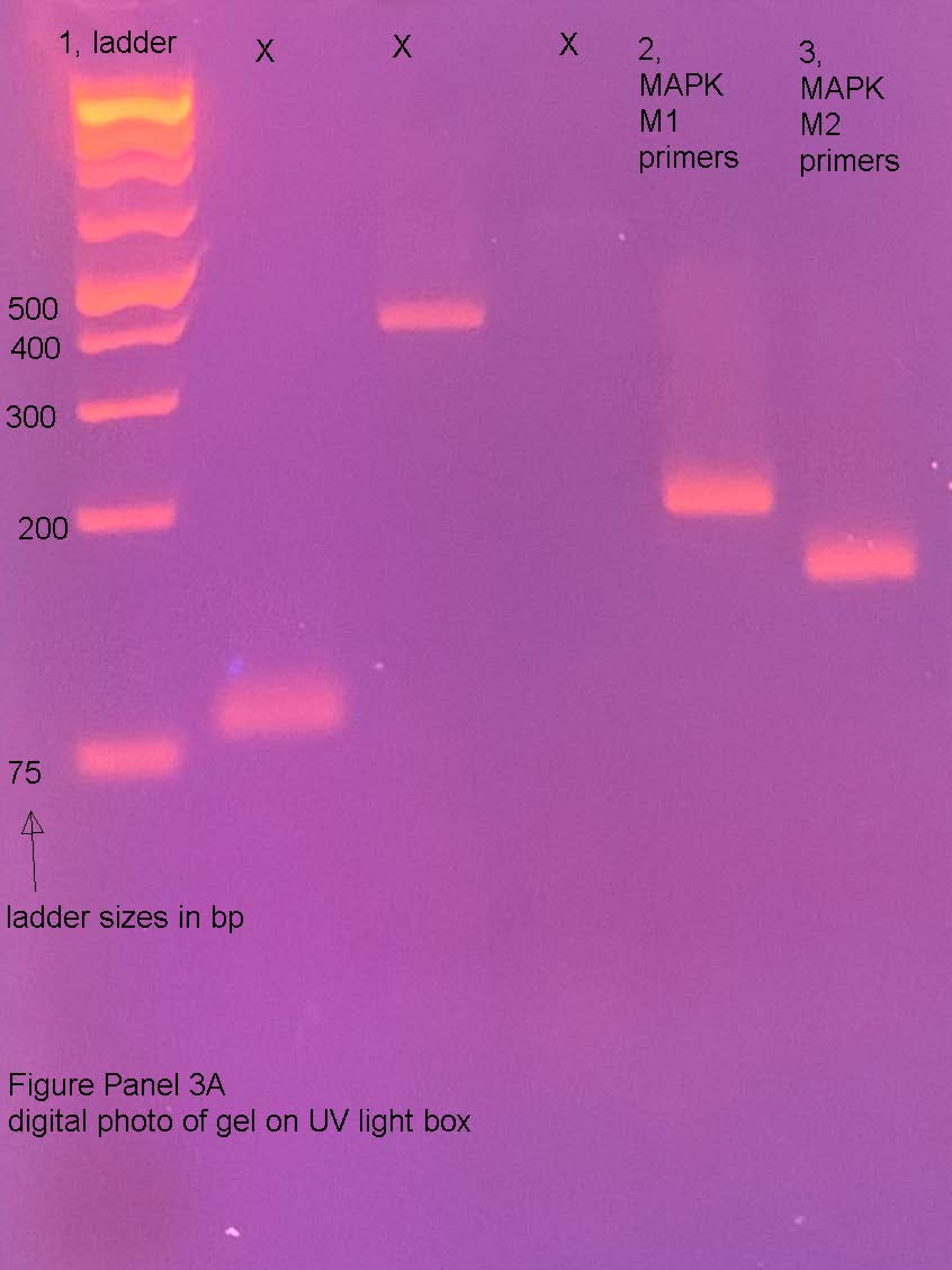


Figure Panel 3A
digital photo of gel on UV light box

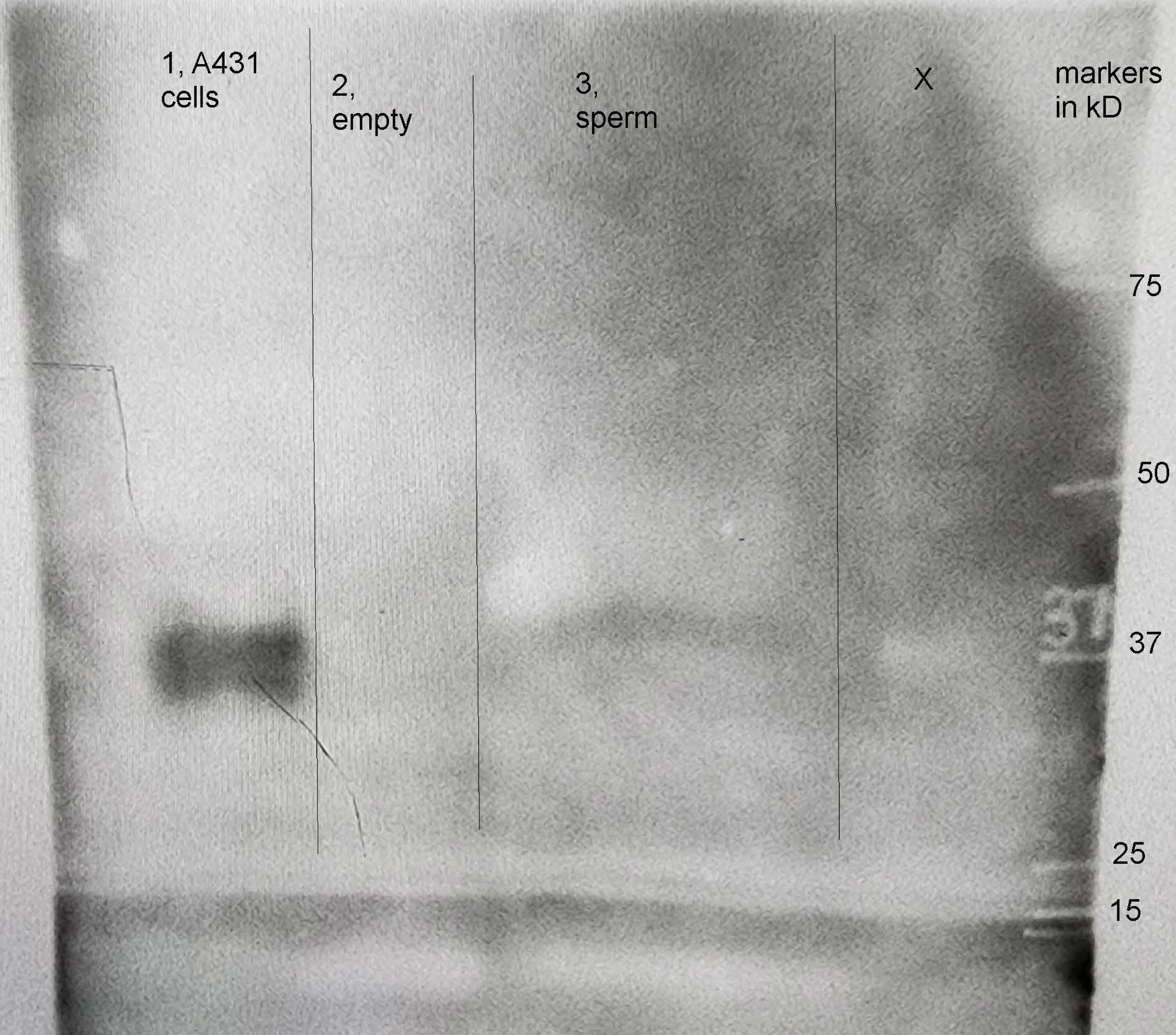


Figure Panel 3B
digital photo of exposed hyperfilm
Note - position of each MWM can be seen as light horizontal line
that had been marked on the blot
vertical lines show apprriximate boundaris of lanes

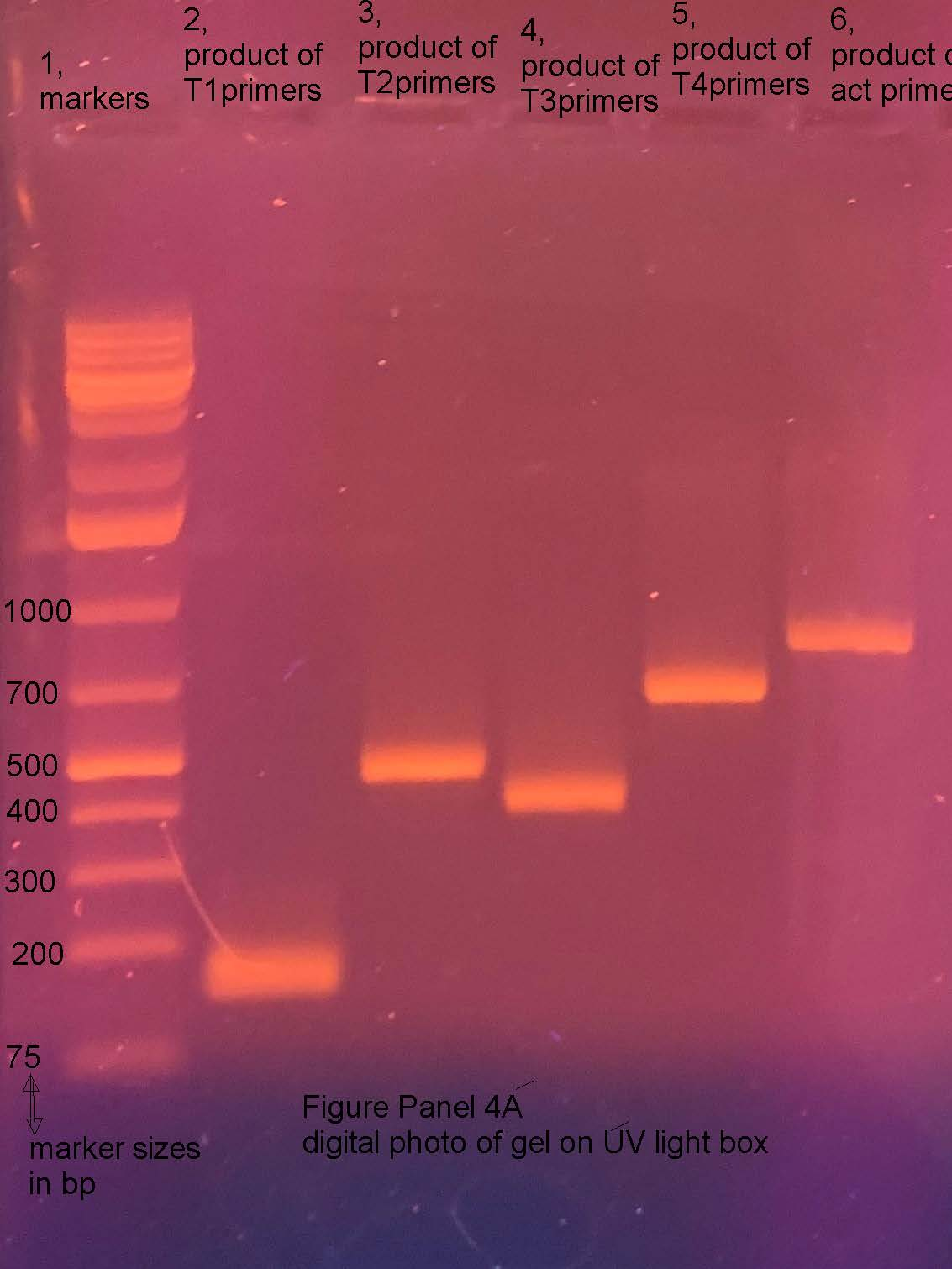


Figure Panel 4A
digital photo of gel on UV light box

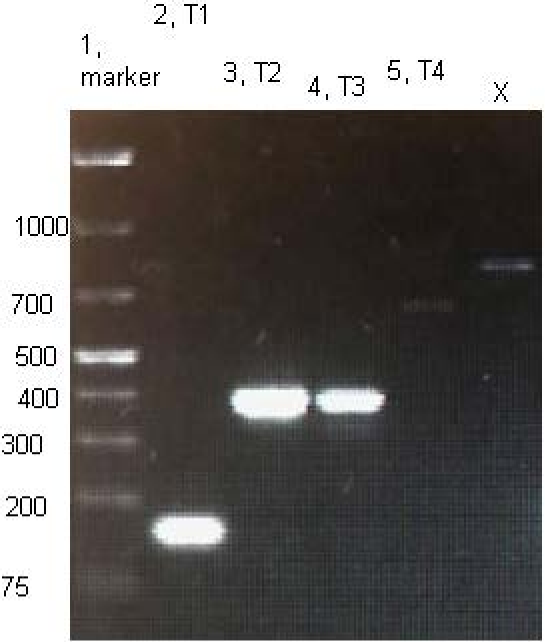


Figure Panel 4B
digital image of thermal print
Note - border added to be able to annotate
